# In vivo efficacy of artemether-lumefantrine and artesunate-amodiaquine for uncomplicated *Plasmodium falciparum* malaria in Malawi, 2014

**DOI:** 10.1186/s12936-016-1281-y

**Published:** 2016-04-26

**Authors:** Magdalena Paczkowski, Dyson Mwandama, Daniel Marthey, Madalitso Luka, Georgina Makuta, John Sande, Doreen Ali, Peter Troell, Don P. Mathanga, Julie Gutman

**Affiliations:** Malaria Branch, Division of Parasitic Diseases & Malaria, Center for Global Health, Centers for Disease Control and Prevention, 1600 Clifton Rd. NE, Mailstop A06, Atlanta, GA 30329-4027 USA; Malaria Alert Centre, University of Malawi College of Medicine, Blantyre, Malawi; United States Peace Corps, Washington, DC USA; National Malaria Control Programme, Ministry of Health, Lilongwe, Malawi; US President’s Malaria Initiative, Malaria Branch, Division of Parasitic Diseases and Malaria, US Centers for Disease Control and Prevention, Lilongwe, Malawi

**Keywords:** *Plasmodium falciparum*, Malawi, Artemether-lumefantrine, Artesunate-amodiaquine

## Abstract

**Background:**

Malaria causes significant morbidity in Malawi, with an estimated 5 million cases in 2014. Artemether-lumefantrine (AL) and artesunate-amodiaquine (ASAQ) are the first- and second-line treatments for uncomplicated malaria, respectively, but emerging resistance threatens their efficacy. In order to understand whether AL and ASAQ remain efficacious for the treatment of uncomplicated *Plasmodium falciparum* malaria in Malawi, a therapeutic efficacy trial was conducted.

**Methods:**

During March–July 2014, febrile children aged 6–59 months with microscopy-confirmed uncomplicated *P. falciparum* malaria (1000–200,000 parasites/μL) were enrolled in a 28-day randomized in vivo efficacy trial at three sites: one each in northern (Karonga), central (Nkhotakota) and southern (Machinga) Malawi. The study was powered to estimate site-specific efficacy for AL and overall efficacy for ASAQ, with 3:1 randomization to AL or ASAQ. Blood was collected for malaria microscopy and molecular testing on days 0–3, 7, 14, 21, and 28. Recrudescence and reinfection were differentiated using polymerase chain reaction (PCR) genotyping of merozoite surface protein. The primary outcome was the PCR-corrected day 28 Kaplan–Meier cumulative success rate.

**Results:**

A total of 452 children were enrolled; 303/338 (89 %) and 98/114 (86 %) reached a study endpoint in AL and ASAQ arms, respectively. All treatment failures occurred after day 3. The day 28 uncorrected cumulative success rate was 97.1 % (95 % confidence interval [CI]: 93.9–100 %) for ASAQ and 76.8 % (95 % CI 72.1–81.5 %) for AL, with 82.5 % (95 % CI 75.4–89.7 %), 69 % (95 % CI 59.9–78.1 %), and 78.2 % (95 % CI 70.2–86.3 %) success in the northern, central, and southern regions, respectively. The day 28 PCR-corrected cumulative success rate was 99 % (95 % CI 97.2–100 %) in the ASAQ arm and 99.3 % (95 % CI 98.3–100 %) in the AL arm, with 98–100 % efficacy in each site.

**Conclusions:**

As evidenced by the day 28 PCR-corrected cumulative success rates, both AL and ASAQ remain efficacious treatments for uncomplicated malaria in Malawi. The lower uncorrected efficacy in the AL arm compared to ASAQ may be explained by the shorter half-life of lumefantrine (3–6 days) compared to amodiaquine (9–18 days). The high reinfection rate suggests that there is a continued need to scale-up effective malaria prevention interventions.

## Background

Malaria is a cause of considerable morbidity and mortality in Malawi, with 5 million cases in 2014. The vast majority of these cases and deaths were caused by *Plasmodium falciparum* [1]. Malaria prevalence among children less than 5 years of age in 2014 was 29 % in the Northern Region, 33 % in the Southern Region, and 36 % in the Central Region [2]. Due to rising resistance to and falling efficacy of sulfadoxine–pyrimethamine, the first-line treatment of uncomplicated malaria from 1993–2007, the National Malaria Control Programme (NMCP) revised the national treatment guidelines in 2007 to recommend artemether-lumefantrine (AL) as the first-line treatment and artesunate-amodiaquine (ASAQ) as a second-line treatment for uncomplicated malaria. Results from recent studies conducted in Malawi and surrounding countries suggest that AL remains efficacious for treatment of uncomplicated *P. falciparum* malaria [3–6].

In 2010, the overall efficacy of AL across six sites in Malawi was 93.4 % [3]. Studies conducted in the neighbouring countries of Tanzania, Zambia, and Mozambique have reported efficacies from 96 to 100 % for both AL and ASAQ [4–6], suggesting that both regimens remain efficacious for treatment of uncomplicated malaria in this region. Given the potential for development of parasite resistance, it is imperative to continue to monitor the efficacy of these regimens [7–9]. The World Health Organization (WHO) recommends routine monitoring of ACT efficacy using standardized in vivo therapeutic efficacy studies and changing treatments when the efficacy of a regimen drops below 90 % [10]. In order to provide the Malawian Ministry of Health with updated estimates of treatment efficacy, a 28-day therapeutic efficacy study of AL and ASAQ for the treatment of uncomplicated *P.falciparum* malaria in children aged six to 59 months was conducted in three sites in Malawi.

## Methods

### Study timing and location

This study was conducted between March and July of 2014 at three district-level hospitals: Karonga District Hospital in the northern region, Nkhotakota District Hospital in the central region, and Machinga District Hospital in the southern region. Malaria transmission is stable throughout the year with a peak in the rainy season, December to April.

### Sample size

The minimum sample size needed to achieve a precision to detect 5 % failures, at a confidence level of 95 %, assuming a drug efficacy of 95 %, was 94 participants per arm. The target sample size was increased to 113 per arm to allow for approximately 15 % loss to follow up. As AL is the first-line treatment and widely used, the study was powered to provide regional estimates for AL efficacy and a country-wide estimate for ASAQ, resulting in a target sample size of 452 participants, 339 in the AL arm and 113 in the ASAQ arm.

### Study procedures

The standard in vivo WHO protocol was used [10]. Children aged 6–59 months presenting to participating hospitals with fever (axillary temperature ≥37.5 °C) or a history of fever in the past 24 h were screened for malaria using a rapid diagnostic test (RDT; Paracheck-Pf^®^, Orchid Biomedical Systems, Goa, India). If the RDT was positive, two thick and one thin blood smears were obtained; an initial rapid reading of the thick smear was used to determine whether the patient met the eligibility criteria for parasitaemia, after enrollment the remaining smears were stained more carefully to obtain more accurate parasite estimates and species. Children who were RDT-positive but ineligible for enrollment were treated with AL according to national guidelines. Children with microscopically detected *P. falciparum* mono-infection with 1000–200,000 asexual parasites/μl were eligible for enrollment provided that they were able to swallow oral medication, had no danger signs, including severe malnutrition, reported no allergies to the study medications, were not taking any medications that could interfere with the study drugs, and the parents/guardians provided informed consent.

After enrollment, children were randomized 3:1 to receive either AL (20 mg artemether/120 mg lumefantrine per tablet, Coartem-D™; Novartis, Basel, Switzerland) or ASAQ (25 mg artesunate/67.5 mg amodiaquine or 50 mg artesunate/135 mg amodiaquine per tablet, Coarsucam™; Sanofi-Aventis, Paris, France). The randomization list was generated for each site using permuted-block randomization in the R (version 3.2.0) blockrand package (R foundation for statistical computing, Vienna, Austria). Study staff in Malawi did not have access to this list. Cards with the treatment assignment were created based on the list and concealed in opaque envelopes that were opened at the time of first administration. Treatment was directly administered with clean water following manufacturer’s prescribed weight-based dosing, twice daily for AL (5–14 kg: 1 tablet; 15–24: 2 tablets; 25–34 kg: 3 tablets; >34 kg: 4 tablets per dose) and daily for ASAQ (4.5–8.9 kg: 1 25 mg/67.5 mg tablet; 9–17.9 kg: 1 50 mg/135 mg tablet; 18–35.9 kg: 2 50 mg/135 tablets; >36 kg: 4 50 mg/135 mg tablets per dose) for 3 days. Participants were observed for 30 min after treatment to monitor for vomiting or other adverse events; those who vomited were administered a second dose and observed for an additional 30 min. Patients who vomited both doses were removed from the study and referred to the hospital for parenteral treatment. Study staff directly observed all three ASAQ doses and all three morning AL doses. Parents or guardians were given the evening AL doses with instructions for administering the drug; parents were asked to verify administration at the visit the following day.

Patients were seen routinely on day 0 (enrollment), days 1, 2, 3, 7, 14, 21 and 28 [10], as well as on any other day when they felt ill. At each visit, a standard symptoms questionnaire and clinical exam was conducted and blood was collected for thick and thin smears and for molecular testing. On days 0, 14 and 28, additional blood was collected for haemoglobin (Hb) testing (Hemocue, Inc, Cypress, CA, USA). On D0 and D7, participants randomized to ASAQ had blood drawn for aspartate aminotransferase (AST) and alanine aminotransferase (ALT) levels.

### Microscopic blood examination

At enrollment, two slides were obtained. The first slide (thick smear only) was stained rapidly, using 10 % Giemsa for 10–15 min, and parasitaemia counted to determine eligibility. In patients deemed eligible for enrollment, the second slide (thick and thin smears) was stained with 2.5–3 % Giemsa for 45–60 min and re-read; the results from the second slide constituted the final enrollment parasitaemia. For all subsequent visits, only the slower stain methodology was used.

Two on-site microscopists independently read the smears according to WHO protocol, each was blinded to the other’s read [10]. If species were discordant or parasitaemia estimates differed by more than 50 % on final study review approximately 1 month after the study completed, slides were read by a third microscopist [10]. Parasites were counted against 200 white blood cells (WBCs) in the thick smear. A minimum of 100 fields were examined to exclude mixed infections; if a mixed infection could not be excluded based on thick smear, the thin smear was examined for confirmation. A total of 1000 WBCs were counted in order to classify a smear as negative; a smear was negative if two microscopists identified it as negative. Parasitaemia was calculated by dividing the number of asexual parasites counted by the number of leukocytes counted, multiplied by an assumed average of 8000 WBCs/μl blood. The final parasitaemia was the geometric mean of the nearest two independent readings.

### Study outcomes

Patients were classified according to the WHO definition of therapeutic responses [10]. Early treatment failure (ETF) was defined as having signs of severe malaria on days 1–3, with parasitaemia on day 2 higher than on day 0 (irrespective of axillary temperature), parasitaemia on day 3 with axillary temperature ≥37.5 °C, or parasitaemia on day 3 ≥25 % of count on day 0. Late treatment failure was divided into two categories: late clinical failure (LCF) and late parasitologic failure (LPF). LCF was defined as danger signs or severe malaria in the presence of parasitaemia on any day between day 4 and day 28 in patients who did not previously meet any of the criteria of ETF or presence of parasitaemia on any day between day 4 and day 28 with axillary temperature ≥37.5 °C or history of fever in patients who did not previously meet any of the criteria of early treatment failure. LPF was defined as presence of parasitaemia on any day between day 7 and day 28 and axillary temperature <37.5 °C in patients who did not previously meet any of the criteria for early treatment failure or late clinical failure. Adequate clinical and parasitologic response (ACPR) was defined as absence of parasitaemia on day 28, irrespective of axillary temperature, in patients who did not previously meet any of the criteria of ETF, LCF, or LPF. Children who met criteria for ETF, LCF, or LPF were treated with the regimen to which they were not randomized and were removed from further study participation. Loss to follow-up occurred when, despite all reasonable efforts, enrolled patients did not attend the scheduled visits and could not be found or located. Every effort was made to schedule a follow-up visit for patients who failed to return to the study site, especially during but also after administration of the study drug. Patients who missed day 1, 2, or 3, or missed more than one visit after day 3 were classified as lost to follow-up. No treatment outcome was assigned to these patients and their last day in the study was the last visit they attended.

### Molecular testing

In order to differentiate between reinfection and recrudescence, DNA samples extracted from paired dried blood spot samples from day 0 and the day of recurrence were subjected to nested PCR for two microsatellite markers, merozoite surface protein 1 (*msp1*) and merozoite surface protein 2 (*msp2*), using WHO recommended PCR procedures [10]. Differences in amplicon length were assessed on a 2 % agarose gel with day 0 and the day of recurrence samples run in adjacent lanes for ease of size comparison.

### Statistical analysis

The primary efficacy endpoint was the day 28-PCR-adjusted cure rate, estimated by Kaplan–Meier analysis. The uncorrected and PCR-corrected Kaplan–Meier cumulative success rates on day 28 were calculated among all participants with any follow-up time; patients were censored when they reached a valid study endpoint, or were lost to follow-up or withdrawn. In addition, the uncorrected and PCR-corrected ACPR on day 28 was calculated among only those participants who reached a valid study endpoint. Where sample sizes were sufficient, t-tests and ANOVA were used to compare means, Wilcoxon and Kruskal–Wallis tests to compare medians and Pearson’s Chi square to compare proportions. P-values less than 0.05 or non-overlapping 95 % confidence intervals (CIs) were considered significant for all statistical tests. Greenwood’s formula was used to calculate 95 % CIs for Kaplan–Meier cumulative success rates. All data analysis was done using SAS version 9.3 (SAS Institute, Carey, North Carolina).

### Ethical considerations

Written, informed consent was obtained from the legal guardian of each study participant. This study was approved by the ethical review boards at the Centers for Disease Control and Prevention (CDC) in Atlanta, GA (protocol #6029) and the Malawi College of Medicine in Blantyre, Malawi (protocol #P.11/13/1486).

## Results

**Fig. 1:**
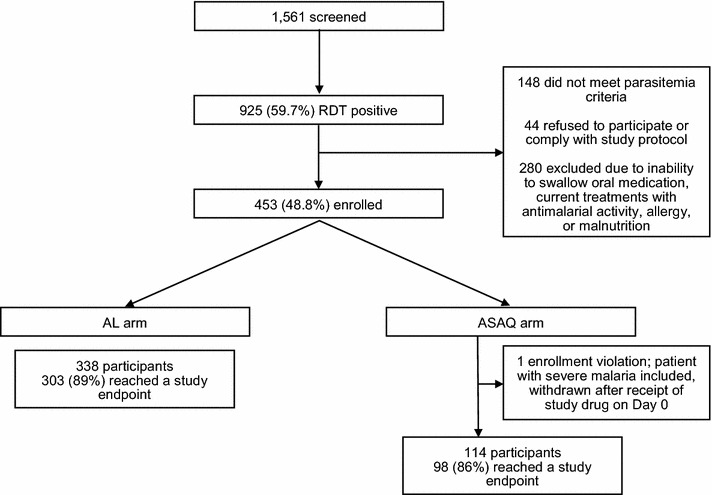
Screening, enrollment, and follow-up of study participants in a therapeutic efficacy study in Machinga, Nkhotakota, and Karonga Districts, in Malawi, 2014

A total of 1561 children were screened; 925 (59.7 %) were RDT positive and, of those, 452 (48.8) were enrolled (Fig. 1). Of those who were RDT positive but not enrolled, 31.3 % did not meet parasitaemia criteria, 9.3 % were unwilling to comply with the study protocol or refused to participate, and the remaining 59.4 % were excluded due to inability to swallow oral medication, taking medication in the past 2 weeks with anti-malarial activity, allergy, or malnutrition. In the AL arm, 338 were enrolled, 332 (97.9 %) completed treatment as assigned, and 303 (89 %) reached a valid study endpoint (29 were lost to follow up). In the ASAQ arm, 114 were enrolled, 110 (95.7 %) completed treatment as assigned, and 98 (86 %) reached a valid study endpoint (12 were lost to follow up).

Characteristics at enrollment were generally similar between both study arms (Table 1). Children randomized to AL were significantly younger and a higher proportion were female compared to children randomized to ASAQ. Insecticide-treated bed net (ITN) use the previous night differed between sites, with 64.2 % of children sleeping under an ITN in Nkhotakota, 89.1 % in Karonga, and 91 % in Machinga. A lower proportion of children in Karonga had anaemia (69 %) compared to Nkhotakota (77 %) and Machinga (88.4 %) (p value <0.0001). Geometric mean parasitaemia was 35,512 parasites/μL (range 1100–394,400) in the AL arm and 30,882 parasites/μL (range 1019–274,908) in the ASAQ arm.

**Table 1:** Participant characteristics at study enrollment in Machinga, Nkhotakota, and Karonga Districts, in Malawi, 2014 (n = 452)

Characteristic	AL				ASAQ
	Total (n = 338)	Site			Total (n = 114)
		Machinga (n = 112)	Nkhotakota (n = 113)	Karonga (n = 113)	
Age, months (median, range)*^, †^	31 (6, 58)	35 (7, 58)	27 (6, 57)	34 (6, 57)	38 (9, 59)
Male, n (%)*	176 (52.1)	67 (59.8)	49 (43.4)	60 (53.1)	45 (39.5)
Participant slept under ITN previous night, n (%)^†, a^	269 (81.5)	101 (91)	70 (64.2)	98 (89.1)	92 (84.4)
Weight (kg) (mean, SD)^†^	11.3 (3.6)	11.9 (3.1)	11.3 (2.2)	10.7 (4.8)	11.7 (3.4)
Haemoglobin (g/dL) (mean, SD)^†^	9.8 (2.3)	9.4 (3.2)	9.7 (1.8)	10.2 (1.5)	10.0 (1.9)
Anaemia (Hb ≤11.0 g/dL)^†^	264 (78.1)	99 (88.4)	87 (77)	78 (69)	84 (73.7)
Parasitaemia (parasites/μL) (geometric mean, range)^†, b^	35,512 (1100, 394,400)	25,700 (1100, 198,000)	39,171 (1393, 394,400)	44,361 (2066, 265,750)	30,882 (1019, 274,908)

All participants were eligible for enrollment based on initial parasitaemia (1000–200,000 parasites/μL); however, 26 (6.1 %) participants had parasitaemia higher than 200,000 parasites/μL according to slide reviews done after study completion

* Significant difference between study arms (p < 0.05); ^†^significant difference between study sites (p < 0.05)

^a^Insecticide-treated bed net

^b^Asexual parasitaemia

### Treatment efficacy

**Fig. 2:**
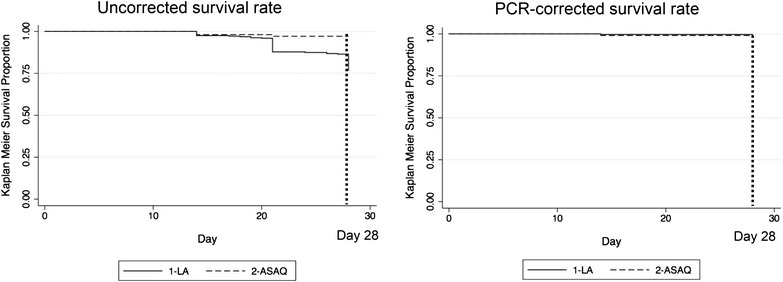
Uncorrected and PCR-corrected Kaplan–Meier cumulative survival rate, Malawi, 2014

There were no ETFs in either arm (Table 2). Only one participant remained parasitaemic on day 3. This participant was in the AL arm and had a day 3 parasitaemia of 99.7 parasites/μL, 0.05 % of day 0 parasitaemia (198,000 parasites/μL). In the uncorrected Kaplan–Meier cumulative success rate analysis, most failures in the AL arm occurred on day 21 (Fig. 2) and 76.8 % (95 % CI 72.1–81.5) of participants remained parasite free on day 28. Nkhotakota had the lowest percent of participants who remained parasite free on day 28 (69.0 %; 95 % CI 59.9–78.1), followed by Machinga (78.2 %; 95 % CI 70.2–86.3), and Karonga (82.5 %; 95 % CI 75.4–89.7). In the ASAQ arm 99.0 % (95 % CI 97.2–100.0) of participants remained parasite free, with the first two failures observed on day 14 (Fig. 2). Uncorrected ACPR results were similar to uncorrected Kaplan–Meier estimates. In the AL arm the uncorrected ACPR was 76.2 % (95 % CI 71–80.9) and in the ASAQ arm, the uncorrected ACPR was 96.9 % (95 % CI 94.5–100.0).

**Table 2:** Participant response to treatment over 28-day follow-up among participants in a therapeutic efficacy study in Machinga, Nkhotakota, and Karonga Districts in Malawi, 2014 (n = 452)

Estimate	AL				ASAQ
	Total (n = 338)	Site			Total (n = 114)
		Machinga (n = 112)	Nkhotakota (n = 113)	Karonga (n = 113)	
Participants lost to follow-up, n (%)	35 (10.4)	14 (12.5)	15 (13.3)	6 (5.3)	16 (14)
Treatment failure, n (%)					
Early	0 (0)	0 (0)	0 (0)	0 (0)	0 (0)
Late	72 (23.8)	22 (22.5)	31 (31.6)	19 (17.8)	3 (3.1)
Day of failure, median (range)	21 (14, 28)	24.5 (14, 28)	21 (14, 28)	21 (14, 28)	14 (14, 21)
Reinfection*	70 (23.1)	22 (22.5)	31 (31.6)	17 (15.9)	2 (2)
Recrudescence	2 (0.66)	0 (0)	0 (0)	2 (1.9)	1 (1)
Day 3 clearance, % (95 % CI)^a^	99.7 (98.3–100)	99.1 (94.5–100)	100 (96.7–100)	100 (96.7–100)	100 (96.7–100)
ACPR, % (95 % CI)^b^					
Uncorrected	76.2 (71–80.9)	77.6 (68–83.4)	68.4 (58.2–77.4)	82.2 (73.7–89)	96.9 (91.3–99.4)
PCR-corrected	99.3 (97.6–99.9)	100 (96.3–100)	100 (96.3–100)	98.1 (93.4–100)	99 (94.5–100)
Kaplan–Meier survival rate on day 28, % (95 % CI)^c^					
Uncorrected	76.8 (72.1–81.5)	78.2 (70.2–86.3)	69 (59.9–78.1)	82.5 (75.4–89.7)	97.1 (93.9–100)
PCR-corrected	99.3 (98.3–100)	100^d^	100^d^	98.0 (95.3–100)	99.0 (97.2–100)

* Significant difference between study sites (p < 0.05)

^a^Percent day 3 clearance was estimated only among participants still enrolled in the study on day 3

^b^Adequate clinical and parasitologic response (ACPR) was estimated only among participants who reached a valid study endpoint

^c^The Kaplan–Meier cumulative survival rate estimate included all study participants who contributed person-days during the 28-day follow-up

^d^For Kaplan–Meier cumulative survival rates of 100 %, confidence intervals were not estimated

Using PCR-corrected data, of the 72 LTFs in participants randomized to AL, 70 were classified as reinfections and two as recrudescences. The median time to reinfection was 21 days (Fig. 2). One recrudescence occurred on day 14 and the other on day 28. Nkhotakota (31.6 %) had a significantly higher proportion of reinfections compared with Machinga (22.4 %) and Karonga (15.9 %). In the ASAQ arm, three LTFs occurred; two were reinfections that occurred on day 14 and 21, and one was a recrudescence that occurred on day 14 (Fig. 2). The PCR-corrected Kaplan–Meier cure rate was 99.3 % (95 % CI 98.3–100) and similar across sites in the AL arm and 99.0 % (95 % CI 97.2–100) in the ASAQ arm.

Of the 452 study participants, 26 (6.1 %) had parasitaemia greater than 200,000 parasites/μL. These participants were eligible for study inclusion based on the initial screening of the thick smear but were later found to have an enrollment parasitaemia higher than 200,000 parasites/μL according to a final study review. All analyses were performed both with (Tables 1 and 2) and without these participants (Tables 3 and 4); the results were not substantially different between the analyses.

**Table 3:** Participant characteristics at study enrollment in Machinga, Nkhotakota, and Karonga Districts, in Malawi, 2014, restricted to participants with parasitemia at enrollment <200,000 parasites/μL (n = 426)

Characteristic	AL				ASAQ
	Total (n = 321)	Site			Total (n = 105)
		Machinga (n = 112)	Nkhotakota (n = 104)	Karonga (n = 105)	
Age, months (median, range)*^, †^	31 (6, 58)	35 (7, 58)	27 (6, 57)	32 (6, 57)	38 (9, 59)
Male, n (%)*	170 (53)	67 (59.8)	46 (44)	57 (54.3)	43 (41)
Participant slept under ITN previous night, n (%)^†, a^	258 (82.2)	101 (91)	64 (64)	93 (90.3)	85 (85)
Weight (kg) (mean, SD)	11.3 (3.5)	11.9 (3.1)	11.3 (2.2)	10.7 (4.8)	11.6 (3.5)
Haemoglobin (g/dL) (mean, SD)	9.7 (2.4)	9.4 (3.2)	9.7 (1.8)	10.1 (1.6)	9.9 (1.8)
Anaemia (Hb ≤ 11.0 g/dL)^†^	250 (78)	99 (88.4)	78 (75)	73 (70)	79 (75.2)
Parasitaemia (parasites/μL) (geometric mean, range)^b^	31,888 (1098, 198,789)	25,700 (1100, 198,000)	32,860 (1393, 198,789)	40,135 (2066, 199,187)	25,952 (1019, 197,701)

All participants were eligible for enrollment based on initial parasitaemia (1000–200,000 parasites/μL); however, 26 (6.1 %) participants had parasitaemia higher than 200,000 parasites/μL according to slide reviews done after study completion

* Significant difference between study arms (p < 0.05); ^†^significant difference between study sites (p < 0.05)

^a^Insecticide-treated bed net;

^b^Asexual parasitaemia.

**Table 4:** Response to treatment over 28-day follow-up among participants with enrollment parasitemia <200,000 parasites/μL in a therapeutic efficacy study in Machinga, Nkhotakota, and Karonga Districts in Malawi, 2014 (n = 426)

Estimate	AL				ASAQ
	Total (n = 321)	Site			Total (n = 105)
		Machinga (n = 112)	Nkhotakota (n = 104)	Karonga (n = 105)	
Participants lost to follow-up, n (%)	33 (10.3)	14 (12.5)	14 (13.5)	5 (4.8)	15 (14.3)
Treatment failure, n (%)					
Early	0 (0)	0 (0)	0 (0)	0 (0)	0 (0)
Late*	70 (24.3)	22 (22.5)	30 (33.3)	18 (18)	2 (2.2)
Day of failure, median (range)	21 (14, 28)	24.5 (14, 28)	21 (14, 28)	21 (14, 28)	17.5 (14, 21)
Reinfection*	68 (23.6)	22 (22.5)	30 (33.3)	16 (16)	1 (1.1)
Recrudescence	2 (0.7)	0 (0)	0 (0)	2 (2)	1 (1.1)
Day 3 clearance, % (95 % CI)^a^	99.7 (97.5–100)	99.1 (94.5–100)	100 (96.5–100)	100 (96.6–100)	100 (96.6–100)
ACPR, % (95 % CI)^b^					
Uncorrected	75.7 (70–80.5)	77.6 (68–83.4)	67.7 (56–76.3)	82 (73.1–89)	97.8 (92.2–99.7)
PCR-corrected	99.3 (97.5–99.9)	100 (96.3–100)	100 (96–100)	98 (93–100)	99 (94–100)
Kaplan–Meier survival rate on day 28, % (95 % CI)^c^					
Uncorrected	76.3 (71.4–81.2)	78.2 (70.2–86.3)	67.4 (58–77.1)	82.2 (74.8–89.7)	97.9 (95–100)
PCR-corrected	99.3 (98.3–100)	100^d^	100^d^	97.9 (94.9–100)	99.0 (96.9–100)

* Significant difference between study sites (p < 0.05)

^a^Percent day 3 clearance was estimated only among participants still enrolled in the study on day 3

^b^Adequate clinical and parasitologic response (ACPR) was estimated only among participants who reached a valid study endpoint

^c^The Kaplan–Meier cumulative survival rate estimate included all study participants who contributed person-days during the 28-day follow-up

^d^For Kaplan–Meier cumulative survival rates of 100 %, confidence intervals were not estimated

### Haemoglobin results

**Fig. 3:**
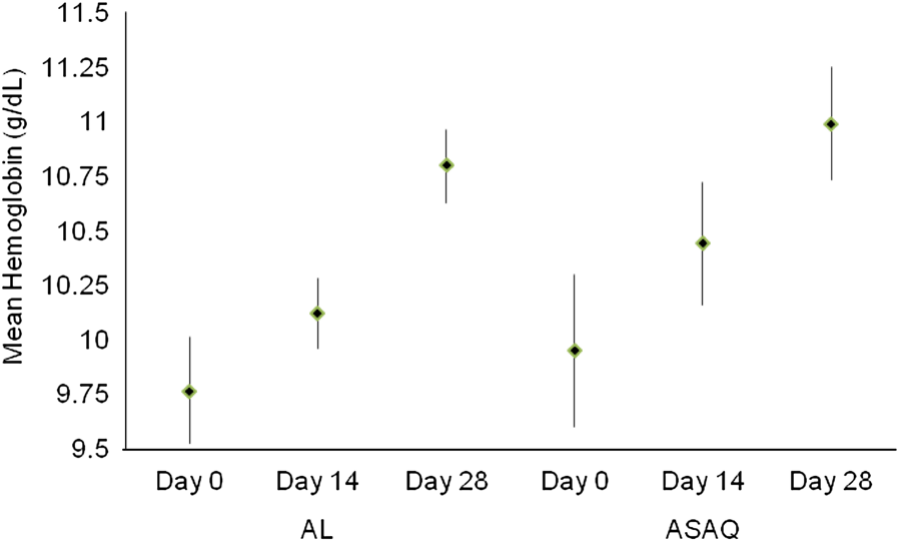
Day 0, day 14, and day 28 mean haemoglobin (Hb) levels among participants randomized to AL and ASAQ, Malawi, 2014

Of those randomized to AL, average Hb levels increased from 9.8 g/dL (95 % CI 9.5–10.0) measured on day 0 to 10.12 g/dL (95 % CI 10.0–10.3) on day 14, reaching 10.8 g/dL (95 % CI 10.6–11.0) by the last visit on day 28 (Fig. 3); this represented a significant increase in the mean Hb levels between day 0 and day 28 (1.2 g/dL, SD 1.6, p < 0.0001). Average Hb levels among ASAQ patients also increased, from 10.0 g/dL (95 % CI 9.6–10.3) on day 0 to 10.4 g/dL (95 % CI 10.2–10.7) on day 14, with a mean value of 11.0 g/dL (95 % CI 10.7–11.3) on day 28 (a 9.5 % compared to day 0); the mean difference in Hb levels between days 0 and 28 was statistically significant (p < 0.0001). Mean differences in Hb levels between days 0 and 28 were not significantly different between study arms.

### Safety and tolerability

Overall, both regimens were well tolerated. Seven patients in the AL arm reported non-compliance with the evening dose; three of these patients subsequently failed therapy. In the AL arm, 21 (6.2 %) of participants vomited at least one dose; four patients (1.2 %) vomited the repeat dose and were withdrawn. In the ASAQ arm, 12 (10.5 %) vomited at least one dose; 1 (0.9 %) vomited the repeat dose and were withdrawn. No significant differences in vomiting the first or second dose were found between study arms. All patients who vomited second doses of either treatment were given rescue treatment. AEs during drug administration were infrequent in both arms, with a maximum of 5 % of patients reporting an AE on day 1, 2, or 3. The most commonly reported AEs were cough, fever, and upper respiratory tract infection. There were a total of four SAEs. One participant in the ASAQ arm was treated with parenteral quinine on Day 1 for a worsening clinical picture. Unfortunately, blood was not drawn for a parasitaemia estimate. Three participants (two in AL arm and one in ASAQ arm) were hospitalized for treatment of pneumonia; none of these were thought to be caused by the study drug.

### Liver function tests

**Fig. 4:**
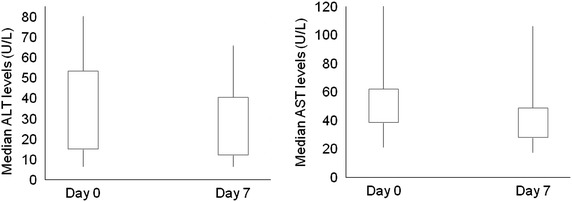
Aspartate transaminase (AST)* and alanine transaminase (ALT)* levels on day 0 and day 7 among participants randomized to ASAQ^†^, Malawi, 2014. *Normal range: ALT: 0–50 U/L; AST: 0–60 U/L. ^†^ALT and AST data at both time points available for 28 and 30 participants, respectively. ALT: Day 0 median 42.3 U/L (range: 6.4 U/L, 80.3 U/L; Interquartile range (IQR): 15 U/L, 53.2 U/L); Day 7 median 16.6 U/L (range: 6.5 U/L, 65.7 U/L; IQR: 12.1 U/L, 40.6 U/L); median difference between day 0 and day 7: −9.4 U/L, p = 0.009. AST: Day 0 median 50.6 U/L (range: 21.2 U/L, 122.6 U/L; IQR: 38.8 U/L, 61.9 U/L); Day 7, median 37.1 U/L (range: 17.6 U/L, 106 U/L; IQR: 27.9 U/L, 48.7 U/L); median difference between day 0 and day 7: −13.4 U/L; p = 0.002. No participants had AST or ALT values more than 2.5 times the upper limit of normal at any time

Among ASAQ participants, the median day 0 ALT was 42.3 U/L (range 6.4, 80.3 U/L) and the median AST was 50.6 U/L (range 21.2, 122.6 U/L) (Fig. 4). By day 7, median ALT levels decreased to 16.6 U/L (range: 6.5, 65.7 U/L) and median AST to 37.1 U/L (range 17.6, 106 U/L). The median differences between day 7 and day 0 were statistically significant for both ALT (median difference: −9.4 U/L, p = 0.009) and AST (median difference: −13.4 U/L; p = 0.002) levels (Fig. 4). No participants had AST or ALT values more than 2.5 times the upper limit of normal at any time (normal range: ALT: 0–50 U/L; AST: 0–60 U/L).

## Discussion

Both AL and ASAQ remain highly efficacious and well tolerated treatments for uncomplicated *P. falciparum* malaria in children age 6–59 months in Malawi, with PCR-corrected ACPRs well above the 90 % WHO threshold for both treatments, and high day 3 parasite clearance for both treatments. The uncorrected efficacy of AL was significantly lower than that of ASAQ, with significant differences in the numbers of reinfections between sites. This is likely due to the shorter half-life of AL compared to ASAQ, resulting in a shorter period of post-treatment prophylaxis.

Overall, the corrected efficacies for both AL and ASAQ are consistent with findings from other studies conducted in Malawi and elsewhere in the region [3, 5, 11–13]. In a therapeutic efficacy study conducted in 2010 in six study sites across Malawi, the combined PCR-corrected ACPR for AL was 93.4 % [3]. While the earlier study in Malawi detected five ETFs compared to zero in this study, day 3 parasite clearance was 99.1 %, comparable to the 99.7 % clearance reported here. Taken together, the results from the 2010 study and this study are suggestive of continued, high efficacy of AL. Although there are no previously published reports of ASAQ efficacy in Malawi, results reported here are in line with studies of ASAQ in children 6–59 months from neighbouring countries, including Mozambique, Uganda, Kenya, and Madagascar, which have reported efficacy from 90 to 99.6 % [5, 11–14].

The lower uncorrected efficacy in the AL arm can likely be explained in part by the shorter half-life of lumefantrine (3–6 days) compared to amodiaquine (6–18 days) [15, 16]. In addition, the recent preferential use of AL over ASAQ may have selected for parasites with greater resistance to AL. Although testing for molecular markers of resistance was not performed in this study, a recent study in Uganda also found a significantly higher rate of reinfections in children treated with ASAQ compared to AL, with a greater proportion of isolates demonstrating genetic polymorphisms consistent with resistance to ASAQ than to AL, suggesting that parasite susceptibility to the drugs may also be contributing [14].

While there were differences in uncorrected AL efficacy between sites, these were not statistically significant. However, this study was not powered to detect differences by site, and these differences may have been significant in an appropriately powered study. Reinfections did differ by site, with significantly more reinfections in Nkhotakota compared to the other sites. The higher number of reinfections might be related to lower reported use of ITNs among children sampled from Nkhotakota (64.2 %) compared to those from Machinga (91 %) and Karonga (89.1 %). Alternatively, the higher number of reinfections might reflect a higher transmission intensity in that study site, although malaria prevalence estimates from the 2014 Malaria Indicator Survey did not show large differences between sites (36 % for Nkhotakota, 33 % for Machinga, and 29 % for Karonga) [2]. This suggests a need for continued implementation and expansion of malaria control measures across Malawi.

As expected, haemoglobin levels among children randomized to both treatments improved significantly over the 28-day follow-up. Both AL and ASAQ were well-tolerated and safe, as has been previously reported [12, 17–19]. There were no drug related SAEs. There was no evidence for drug related liver toxicity in patients taking ASAQ. In fact, the median AST and ALT were both significantly lower at day 7 than at enrollment. Prevalence of vomiting following treatment was similar to findings from other studies in the region, finding that 0–8.3 % vomited following AL treatment and 2.1–10.6 % vomited following ASAQ [5, 20–22].

This therapeutic efficacy study had several limitations. First, several participants with a final parasitaemia greater than 200,000 parasites/μl were included, as these patients were deemed eligible according to the initial microscopy reading. Treating these participants with AL or ASAQ could have increased the risk of delayed parasite clearance and recrudescence as parasite biomass might be too high to be adequately cleared [23]. Reassuringly, none of these participants showed delayed parasite clearance nor had recrudescent infections, and the study results were not affected by removal of these participants from the analysis. Secondly, the evening dose of AL was not supervised, due to limited resources. Parents were asked at the visit the following morning whether the previous evening’s dose had been taken by the child. All parents reported having administered the dose. This was additionally verified by having the parents return with the empty medication packaging. While it is possible that some parents incorrectly reported having given the dose, the very high efficacy found for AL suggests that this did not significantly affect the study results. Parental self-report of the second dose in lieu of direct observation has been used in other studies, with no significant effect on study outcomes noted [24]. As missed evening doses would increase the risk of recrudescence, the true efficacy of AL would be higher than reported here if a significant number of evening doses were missed. Finally, the study included only 28 days of follow-up, the minimum recommended by WHO for drugs with elimination half-lives of less than 7 days; any additional recurrences beyond this time frame were not captured.

The results of this study demonstrate that AL and ASAQ remain efficacious treatments for uncomplicated *P. falciparum* malaria in Malawi. There is no evidence at this time that a change in regimens is warranted. However, continued monitoring of drug efficacy, following WHO recommendations, is needed. Finally, the relatively high reinfection rates observed during the study period are evidence of the continued need to scale-up the ownership and use of effective malaria prevention interventions, such as ITNs.

## References

[1] WHO (2014). World malaria report.

[2] National Malaria Control Programme (NMCP) [Malawi] and ICF International: (2014). Malawi malaria indicator survey (MIS).

[3] Dambe R, Sande J, Ali D, Chilima B, Dodoli W, Michelo CC (2015). Monitoring the efficacy of artemether-lumefantrine for the treatment of uncomplicated malaria in Malawian children. Malar J.

[4] Hamainza B, Masaninga F, Moonga H, Mwenda M, Chanda-kapata P, Chalwe V (2014). Therapeutic efficacy of artemether-lumefantrine on treatment of uncomplicated *Plasmodium falciparum* mono-infection in an area of high malaria transmission in Zambia. Malar J.

[5] Nhama A, Bassat Q, Enosse S, Nhacolo A, Mutemba R, Carvalho E (2014). In vivo efficacy of artemether-lumefantrine and artesunate-amodiaquine for the treatment of uncomplicated falciparum malaria in children: a multisite, open-label, two-cohort, clinical trial in Mozambique. Malar J.

[6] Shayo A, Buza J, Ishengoma DS (2015). Monitoring of efficacy and safety of artemisinin-based anti-malarials for treatment of uncomplicated malaria: a review of evidence of implementation of anti-malarial therapeutic efficacy trials in Tanzania. Malar J.

[7] Mita T, Tanabe K (2012). Evolution of *Plasmodium falciparum* drug resistance: implications for the development and containment of artemisinin resistance. Jpn J Infect Dis.

[8] Takala-Harrison S, Jacob CG, Arze C, Cummings MP, Silva JC, Dondorp AM (2015). Independent emergence of artemisinin resistance mutations among *Plasmodium falciparum* in Southeast Asia. J Infect Dis.

[9] Wongsrichanalai C, Sibley CH (2013). Fighting drug-resistant *Plasmodium falciparum*: the challenge of artemisinin resistance. Clin Microbiol Infect.

[10] WHO (2009). Methods for surveillance of antimalarial drug efficacy.

[11] Thwing JI, Odero CO, Odhiambo FO, Otieno KO, Kariuki S, Ord R (2009). In-vivo efficacy of amodiaquine-artesunate in children with uncomplicated *Plasmodium falciparum* malaria in western Kenya. Trop Med Int Health.

[12] Yeka A, Lameyre V, Afizi K, Fredrick M, Lukwago R, Kamya MR (2014). Efficacy and safety of fixed-dose artesunate-amodiaquine vs. artemether-lumefantrine for repeated treatment of uncomplicated malaria in Ugandan children. PLoS One.

[13] Zwang J, Olliaro P, Barennes H, Bonnet M, Brasseur P, Bukirwa H (2009). Efficacy of artesunate-amodiaquine for treating uncomplicated falciparum malaria in sub-Saharan Africa: a multi-centre analysis. Malar J.

[14] Yeka A, Kigozi R, Conrad MD, Lugemwa M, Okui P, Katureebe C (2016). Artesunate/amodiaquine versus artemether/lumefantrine for the treatment of uncomplicated malaria in Uganda: a randomized trial. J Infect Dis.

[15] Makanga M, Krudsood S (2009). The clinical efficacy of artemether/lumefantrine (Coartem). Malar J.

[16] Orrell C, Little F, Smith P, Folb P, Taylor W, Olliaro P (2008). Pharmacokinetics and tolerability of artesunate and amodiaquine alone and in combination in healthy volunteers. Eur J Clin Pharmacol.

[17] Falade C, Makanga M, Premji Z, Ortmann CE, Stockmeyer M, de Palacios PI (2005). Efficacy and safety of artemether-lumefantrine (Coartem) tablets (six-dose regimen) in African infants and children with acute, uncomplicated falciparum malaria. Trans R Soc Trop Med Hyg.

[18] Olliaro P, Mussano P (2003). Amodiaquine for treating malaria. Cochrane Database Syst Rev.

[19] Schellenberg D, Kahigwa E, Drakeley C, Malende A, Wigayi J, Msokame C (2002). The safety and efficacy of sulfadoxine-pyrimethamine, amodiaquine, and their combination in the treatment of uncomplicated *Plasmodium falciparum* malaria. Am J Trop Med Hyg.

[20] Egunsola O, Oshikoya KA (2013). Comparative safety of artemether-lumefantrine and other artemisinin-based combinations in children: a systematic review. Malar J.

[21] Four Artemisinin-Based Combinations Study Group (2011). A head-to-head comparison of four artemisinin-based combinations for treating uncomplicated malaria in African children: a randomized trial. PLoS Med.

[22] Michael OS, Gbotosho GO, Folarin OA, Okuboyejo T, Sowunmi A, Oduola AM (2010). Early variations in plasmodium falciparum dynamics in Nigerian children after treatment with two artemisinin-based combinations: implications on delayed parasite clearance. Malar J.

[23] WHO (2014). Guidelines for the treatment of malaria.

[24] Plucinski MM, Talundzic E, Morton L, Dimbu PR, Macaia AP, Fortes F (2015). Efficacy of artemether-lumefantrine and dihydroartemisinin-piperaquine for treatment of uncomplicated malaria in children in Zaire and Uige Provinces, Angola. Antimicrob Agents Chemother.

